# The phototactic rhythm of pests for the Solar Insecticidal Lamp: A review

**DOI:** 10.3389/fpls.2022.1018711

**Published:** 2023-01-19

**Authors:** Heyang Yao, Lei Shu, Fan Yang, Yinghao Jin, Yuli Yang

**Affiliations:** ^1^ College of Engineering, Nanjing Agricultural University, Nanjing, China; ^2^ College of Artificial intelligence, Nanjing Agricultural University, Nanjing, China; ^3^ School of Engineering, University of Lincoln, Lincoln, United Kingdom; ^4^ School of Mathematics and Statistics, Jiangsu Normal University, Xuzhou, China

**Keywords:** phototactic pest, phototaxis behavior, solar insecticidal lamp (SIL), phototactic rhythm of pests, intelligent pest management

## Abstract

Pest management has been a great challenge since the beginning of human agricultural activities. Since the 1930s, chemical pesticide control has been a major control technology that can solve some of the pest problems in agricultural production. Still, it is harmful to food safety and the ecological environment. Meanwhile, the extensive use of chemical pesticides may lead to the rapid development of pest resistance. Because of the advantages of low cost, eco-friendly advantage, and low side effects, Solar Insecticidal Lamp (SIL) as the main physical control technology has been widely used for pest management in agricultural production in China. Owing to the phototaxis of pests, they have a phototropic rhythm during the nighttime. We can adjust the SIL insecticidal time according to the phototropic rhythm of pests. The purpose of this paper is to provide a comprehensive review of the pest phototactic rhythm in a selection of 24 pest species. It is the first comprehensive survey on the phototactic rhythm of pests and the time segments of this survey are accurate to the hour. The phototactic rhythm of pests are investigated in two different varieties of crops: 1) food crops and 2) economic crops. We also discuss and analyze the various factors (e.g., meteorological conditions, insecticidal devices, physiological states and others) that affect the changing phototactic rhythm of pests. Finally, we highlight some open research challenge issues and future directions.

## 1 Introduction

Throughout the history of human agriculture, pest outbreak not only cause a disastrous impact on agricultural production but also lead to social instability. Pest management has become a major challenge issue (Affrin et al., 2017; Kim et al., 2018; Isman, 2019). As early as the 20th century, (Zhao, 1992) had proposed that eco-friendly methods (including Physical methods) for pest management are a vital direction for future development. Among them, solar insecticidal lamp (SIL) as a physical method plays an important role in pest management because of non-polluting characteristics (Yang et al., 2020; Wang et al., 2020). Currently, SILs are widely adopted for pest management in agricultural fields (Lin, 2015). During the daytime, SIL harvests solar energy through solar panels and stores the energy. During the nighttime, phototropic pests are attracted to the SIL and contact the high-voltage mesh, which kills them by releasing a high-voltage pulse current. Although there are many advantages of SIL, there are also shortcomings. The current traditional SIL mainly adopts the remote time method to control the SIL’s insecticide working time, lacking intelligent energy management.

With the development of integration technologies and communication technologies, the Internet of Things (IoTs) are widely used in smart cities, and smart agriculture (Alsamhi et al., 2019a; Alsamhi et al., 2019b). The integration of SIL and IoTs technology easily forms a new type of agricultural IoT technology - Solar Insecticide Lamp Internet of Things (SIL-IoTs) (Li et al., 2019). SIL-IoTs transmit the high-voltage discharge pulse and status information of the SIL’s current location to the terminal in real time *via* a wireless communication module. The terminal estimates the density of pests in the region at different time slots based on the number of high-voltage pulses released by the SIL. The pests exhibit different phototropic rhythms due to the different times of nighttime outbreaks. When SIL is turned on for a long time, time slots with high pest density consume more energy. The main energy consumption of SIL includes three main aspects: 1) Lamp energy consumption, 2) Insecticide energy consumption and 3) WSN energy consumption. For the same time slots of insecticide work with SIL turned on, the energy utilization is higher for time slots with high pest density and lower for time slots with lower pest density. Therefore, when the pest density is low or even no pests, SILs with the same duration of lamp turn-on will result in low energy utilization. When the density of pests is high, the SIL will miss the best time to kill pests due to the lack of energy. When SIL energy is insufficient and pest density is low, the energy consumption of SIL can be reduced by turning off the lamp early to stop the insecticidal task. Since the pest densities in different time slots can lead to different forms of pest phototropic rhythms. To improve the energy utilization of SIL, it is necessary to adjust the insecticidal work time of SIL according to the pest phototropic rhythm.

Although some progress has been achieved in the study of pest phototropic rhythms, a detailed and systematic summary of the latest research results in this field has not been carried out. Hence, a comprehensive summary of the phototropic rhythm of pests in different crops is necessary to improve the energy utilization rate and insecticidal effect of SIL.

### 1.1 Motivation

The three motivations for this paper are as follows:

- To summarize the phototactic rhythm of pests in different crops (e.g., food crops and economic crops).
- To explore the various factors that affect the phototactic rhythm of pests in different crops (e.g., meteorological conditions, insecticidal devices, physiological states and others).
- To develop technical support for future research and development of pest management equipment.

### 1.2 Contribution

In this paper, we present a comprehensive survey of the motivations, research progress, and applications aimed at achieving the phototactic rhythm of pests. The main contributions of this paper are summarized as follows:

- We present background knowledge on pest phototactic rhythm from the pest management perspective and the current state of research on pest phototactic rhythm as summarized in our work. The application and benefits of pest phototropic rhythms in pest management are discussed in the pest’s characteristics and the smart agriculture challenge issues.
- To deeply understand the phototactic rhythm of pests in different crops, a comprehensive research outline on the phototactic rhythm of pests is summarized. Among them, crops are divided into two main varieties: food crops (e.g., rice, soybean, and maize) and economic crops (e.g., cotton, vegetable, orchards, tea).
- The key factors of pest phototropic rhythms are analyzed to provide data support for the agricultural planters. The key factors include the following four main aspects: meteorological conditions (e.g., temperature, humidity, precipitation, light intensity), insecticidal devices(e.g., wavelength and height of insecticidal devices), physiological states (e.g., sex, age, and dark adaptation), and others (e.g., crop varieties and natural enemies).
- Finally, some research challenges about the pests phototactic rhythm are discussed.

The rest of this paper is organized as follows: In Section II, we analyze the factors that influence the phototactic rhythm of pests. The phototactic rhythm of different crop pests is summarized in Section III. Research challenges and future directions are discussed in Section IV. Finally, we conclude in Section V.

## 2 Related works

**Figure 1:**
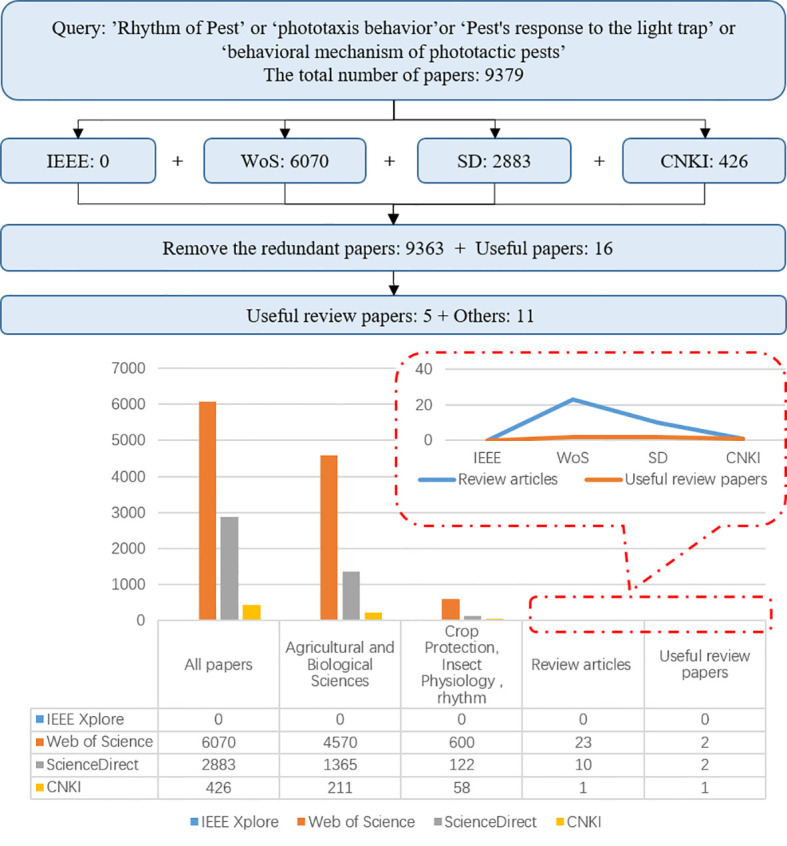
The number of articles among the corresponding reviews.

To investigate related works about the phototactic rhythm of pests, we search four digital databases: 1) IEEE Xplore (IEEE), 2) Web of Science (WoS), 3) Science Direct (SD), and 4) China National Knowledge Infrastructure (CKNI). As a result, a total of 187 survey papers were found at the initial sample stage by searching keywords, such as ‘phototactic rhythm of pests’, ‘phototaxis behavior’, ‘pest’s response to the light trap’, and ‘behavioral mechanism of phototactic pests’. However, the majority of those studies are mainly about biological sciences. Thus, 16 papers (including five survey papers) highly relevant to phototropic rhythms are selected in **Figure 1**.

As depicted in **Table 1**, only (Anat and Byers, 2021) and (Yang et al., 2020) discussed the phototactic rhythm of pests, while other researchers (Saunders and Bertossa, 2011; Deguine et al., 2015; Gottlieb, 2019) mainly explored the factors affecting pests. Although (Anat and Byers, 2021) and (Yang et al., 2020) described the phototropic rhythm, they did not detail the hourly phototropic rhythm of pests throughout the night. Thus, the comprehensive research on the phototropic rhythm of pests is essential.

**Table 1:** Investigation and comparison of the phototactic rhythm of pests.

Paper	Crop classification	Single pest	Multiple pests	Impact factors	Phototactic rhythm of pests	Precision to hour	All the night
Our survey	✓		✓	✓	✓	✓	✓
(Anat and Byers, 2021)			✓	✓	✓		✓
(Yang et al., 2020)			✓	✓	✓		
(Gottlieb, 2019)			✓	✓			
(Deguine et al., 2015)	✓	✓		✓			
(Saunders and Bertossa, 2011)			✓	✓			

"✓" means that the contents of this column are described by this literature.

**Figure 2:**
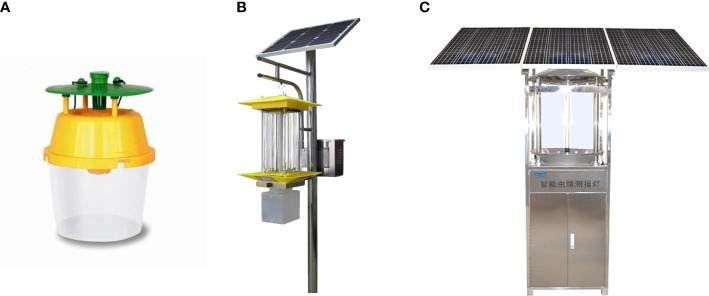
Three types of pest monitoring devices. **(A)** Trap lamp, **(B)** Insecticidal lamp, **(C)** Pest monitoring lamp.

The application of SILs to trap and kill pests has become an important part of pest management. Currently, there are mainly three types of devices (trap lamps, SILs, and pest monitoring lamps) for pest management, as shown in **Figure 2**. Generally speaking, trap lamps are one of the most common insecticide devices to attract pests. Insecticide lamps can effectively attract and kill pests based on their phototactic characteristics. The price of an insecticidal lamp varies with its function. **Table 2** shows that the price of lamp devices fluctuates between 2.8 and 20891.4 dollars. Compared with the above two lamps, the pest monitoring lamp is a practical new tool that uses photoelectric technology to achieve automatic trapping, and monitoring. Although pest monitoring lamps have the advantages of high accuracy and intelligence, they are expensive.

**Table 2:** Price range of different products.

The type of product	Range of prices/Dollar	Average price/Dollar
Trap lamp	1.4 - 5.6	2.8
Frequency-vibrancy insecticidal lamp	4.2 - 390	24.4
Air suction insecticidal lamp	30.6 - 947.1	428.2
Impact insecticidal lamp	27.6 - 766	455
Water-drowning insecticidal lamp	37.3 - 348.2	150.5
Solar insecticidal lamp	30.6 - 2785.5	423.3
Pest monitoring lamp	6963.8 - 25069.6	20891.4

Therefore, major researches have been conducted with cheaper SILs and trap lamps for experiments, as 103 shown in **Table 3**.

**Table 3:** Summary of research papers on the phototactic rhythm of pests in China.

Paper	Different expression	Serviceable tool	Research method	Food crops			Economic crops			Precision to hour	All the night
				Rice	Maize	Cotton	Vegetable	Tea	Orchards		
(Tu et al., 2018)	Rhythm of pests	LED solar insecticidal lamp	Time control					✓		✓	
(Zhang et al., 2017)	rhythm of pest	Trap lamp	Time control			✓	✓			✓	✓
(Tu et al., 2016)	Rhythm of pests	LED solar insecticidal lamp	Time control						✓	✓	
(Ma, 2016)	Rhythm of pests	Vibration frequency insecticidal lamp	Time control	✓							
(Cai et al., 2016)	Rhythm of pests	Vibration frequency insecticidal lamp	Time control		✓					✓	✓
(Qi et al., 2014)	Rhythm of pests	Search lamp	Time control	✓						✓	✓
(Yang et al., 2012)	Rhythm of pests	Intelligent solar insecticidal lamp	Artificial control	✓	✓		✓			✓	✓
(Wan et al., 2008)	Pests’ rhythm	Vibration frequency insecticidal lamp	Time control				✓			✓	✓
(Gu et al., 2004)	Rhythm of several night active insects	Doublewaves trapping lamp	Artificial control			✓				✓	✓
(Chen et al., 2001)	Pests response to lamp trap	Trap lamp	Optical control	N/A	N/A	N/A	N/A	N/A	N/A	✓	✓
(Zhao, 1992)	Behavioral mechanism of phototactic pests	Black light lamp	Artificial control	✓							

"✓" means that the contents of this column are described by this literature.

The symbol "N/A" means that the content of this column is not described by this literature, which is not clear whether it contains or specific values.

Since there are few studies on the phototactic rhythm of pests abroad, our paper mainly reviews research papers on the phototactic rhythm of different pests in China. Above researches (Zhao, 1992; Gu et al., 2004; Wan et al., 2008; Qi et al., 2014; Cai et al., 2016; Ma, 2016; Tu et al., 2016; Tu et al., 2018; Yang et al., 2020) have studied the phototactic rhythm of single crop pests. However, only (Yang et al., 2012; Zhang et al., 2017) had investigated the phototropic rhythm of pests in more than two crops. (Yang et al., 2012; Qi et al., 2014) studied the phototropic rhythm of rice pests precisely to one time slot per hour. In addition, (Gu et al., 2004; Wan et al., 2008; Cai et al., 2016; Tu et al., 2016; Tu et al., 2018) also studied the phototropic rhythm of single crop (vegetable, cotton, maize, vineyard, tea garden) pests in hourly time slots, and the phototropic rhythm of pests in these crops showed different forms. Thus, crop varieties have a strong influence on the phototropic rhythm of pests.

## 3 Factors affecting the phototactic rhythm of pests

The phototactic rhythm of pests are influenced by some factors, such as meteorological conditions (e.g., temperature, humidity, precipitation, light intensity), insecticidal devices(e.g., wavelength and height of insecticidal devices), physiological states (e.g., sex, age, and dark adaptation), and others(e.g., crop varieties, and natural enemies) (Steinbauer et al., 2012; Van et al., 2014; Carayon et al., 2014; Affrin et al., 2017; Owens and Lewis, 2018).

### 3.1 Meteorological conditions

Meteorological conditions have a great influence on pest phototropic rhythms, such as temperature, humidity, precipitation, and light intensity.

- ***Temperature*** The phototactic rhythm of the pest varies with the ambient temperature due to its poor ability to maintain and regulate body temperature. Therefore, the temperature has a strong influence on pest growth and phototropic rhythm (Nordstrom and Warrant, 2000). In general, the higher the temperature, the stronger the phototactic rhythm of pests (Saunders, 2014). The temperature can affect the pupil of the pest’s eye, and when the temperature is lower than 20°C, the pest loses the attraction to light. Therefore, low temperatures may reduce the phototactic rhythm of pests (Dang and Chen, 2012). However, the temperature rises, the mortality rate of *Ostrinia Furnacalis* may decreases (Dong, 2016). Hence, the local temperature and humidity have an impact on the growth of *Ostrinia Furnacalis*.
- ***Humidity and Precipitation*** In general, precipitation as a non-biological factor affects the environmental temperature and humidity. Since temperature and humidity tend to cause changes in pest behavior, precipitation has a definite effect on pest phototropic rhythms (Chang et al., 2008), precipitation has a huge impact on the phototropic rhythm of pests. Pests are forced to land when it rains. It is until the rain stops that they take off and fly to the light source.
- ***Light intensity*** Light intensity affects the phototactic rhythm of pests, as the take-off time of the pest is influenced by the duration of the light cycle (a period of day and night) (Saunders, 2013). Phototropic pests prefer the brightness of light intensity (Yu, 2011; Kim et al., 2020). There are differences in the phototropism of pests under different light intensities, and some pests even show opposite phototactic rhythm due to the light intensity variations (Hai et al., 2016). For example, the phototactic rhythm of adult pests increased linearly with the increase of light intensity. However, there was no absence of phototropic behavior in adult pests under low light (Zhang et al., 2009). Generally, the optimum light intensity for the *Helicoverpa Armigera* activity is 0.1 - 0.001 lux, especially under 0.001 lux (Hou et al., 1997). As the light intensity increased, the phototactic rhythm of the pests also increased, but not in proportion.

### 3.2 Types of insecticidal devices

The light source wavelength and height of the insecticidal devices also affect the pest phototactic rhythm in terms of the type of insecticidal device.

- ***Light source Wavelength of the insecticidal devices*** The human eye can perceive wavelengths from 390 to 750 nm. Unlike humans, the sensitive spectrum of pests is mainly concentrated in the 253 - 700 nm (Pan et al., 2021). Thus, pests can perceive different light source wavelengths (Liu et al., 2018). Currently, there are various studies on the phototropism of pests at different wavelengths. For example, the single black lamp has the strongest attraction to *Holotrichia parallela*, while the black lamp and white lamp are stronger than a single black lamp for the *Helicoverpa Armigera*, *Ostrinia Furnacalis*, *Mythimna Sepera*, *Chilo Suppressalis*, and *Cnaphalocrocis Medinalis*. Black lamp and green lamp are the most attractive to *Anomala Corpulenta Motschulsky*. However, *Aphidoidea* and *Agromyzidae* pests have a strong phototropism to yellow and green lamp (Yee, 2015), while silver-gray has a strong repellent effect on the leafminers (Chen et al., 2015). The results think that the phototactic rhythm of pests are the result of the long-term evolution of multiple factors.
- ***Height of the insecticidal devices*** From the perspective of the insecticidal devices, the insecticidal device height also has an effect on the phototactic rhythm of pests. The height of the insecticide device should be determined by the height of the crop. In the major crop areas, the height of the insecticidal lamp should exceed the top of the crop (Zhang et al., 2008). Therefore, the height of insecticidal lamps should be set at 0.7 - 0.8 meters for low crop areas (Wan et al., 2008).

### 3.3 Physiological states of pests

Pests have evolved different visual structures in complex environments. Different wavelengths and other external environments may produce more complex physiological responses to pests, resulting in different forms of their phototropic rhythm of pests. Pests’ physiological state may be determined by their sex, age, and dark adaptation. Therefore, the phototactic rhythm of pests are significantly influenced by their physiological status (Cheng et al., 2011; Kim et al., 2018). Moreover, the physiological state of pests changes with seasons and environment, so the determination of the phototactic rhythm of pests in different seasons may have different results (Andersen, 2006; Do et al., 2012).

- ***Sex and age*** Pests have different phototropism at different developmental stages and phototactic rhythm changes with sex and age. For example, on the first day of the pest’s emergence, females are more phototropic than males. But on the second, fourth, sixth, and twelfth days, the phototactic rhythm of males was about twice as high as that of females (Keil et al., 2001). The phototactic rhythm of *Tortricidae* was highest on the twelfth day of age for females and on the fourth day of age for males in the family*Tortoiseidae*. In general, the phototactic rhythm of turtle pests is most pronounced at dusk (Keil et al., 2001). In addition, pests of different ages respond differently to the same wavelength (Zhang et al., 2016). The light sensitivity of 1 - 5 day old adult of *Spodoptera Litura* is higher (Xu et al., 2014).
- ***Dark adaptation*** The retinal cells of the pest’s eyes contract in a light-dark environment, which tends to cause the pest to take some time to turn to the dark environment. Therefore, the pest’s dark adaptation time is related to the time required for the pest’s eyes to turn to dark adaptation. At night, the phototactic rhythm of pests are also closely related to the pest’s dark adaptation because the phototropism of fully dark-adapted pests is higher than that of other pests (Kim et al., 2019).

### 3.4 In other ways

**Figure 3:**
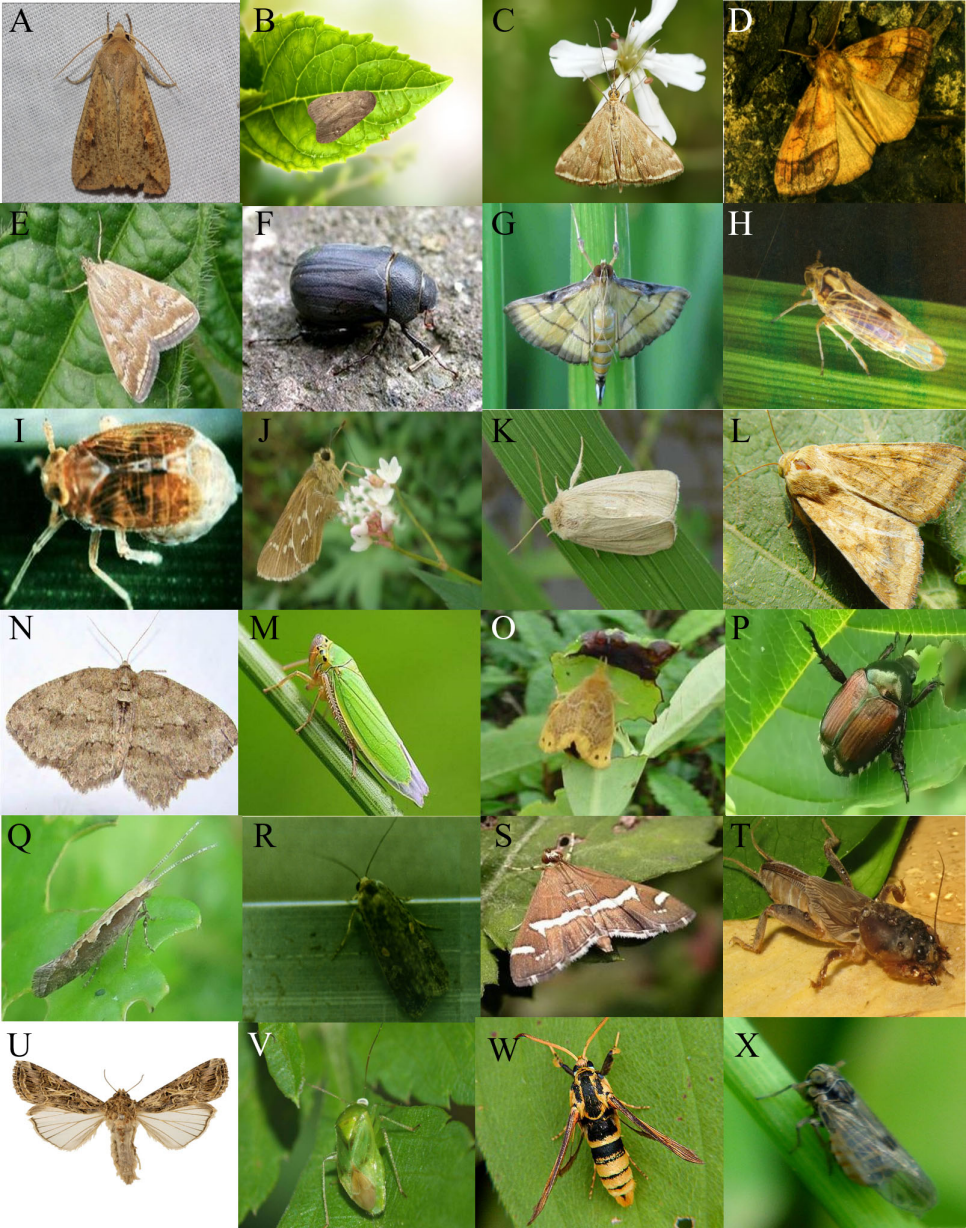
The pictures of pest species. **(A)** Mythimna Seperata, **(B)** Proxenus Lepigone, **(C)** Ostrinia Furnacalis, **(D)** Cifuna Locuples Walker, **(E)** Loxostege Sticticalis, **(F)** Holotrichia Perllela, **(G)** Cnaphalocrocis Medinalis, **(H)** Sogatella Furcifera, **(I)** Nilaparvata Lugens Stal, **(J)** Naranga Aenescens, **(K)** Chilo Suppressalis, **(L)** Helicoverpa Armigera, **(N)** Ectropis Obliqua Hypulina Wehrli, **(M)** Empoasca Pirisuga Matumura, **(O)** Euproctis Pseudoconspersa Strand, **(P)** Anomala Corpulenta Motschulsky, **(Q)** Plutella Xylostella, **(R)** Spodoptera Exigua, **(S)** Spoladea Recurvalis, **(T)** Gryllotalpa Orientalis Burmeister, **(U)** Spodoptera Litura, **(V)** Apolygus Lucorum, **(W)** Paranthrene Regalis Butler, **(X)** Laodelphax Striatellus (The pictures come from Image Database for Agricultural Diseases and Pests Research (IDADP).

- ***Crop varieties*** As some pests feed on twigs, flowers and other nutritional organs for long periods of time, they evolve over time to be phototropic to the pigments in these crops. Therefore, pests are attracted to different crop varieties (Hajong and Varman, 2002). In summary, the phototactic rhythm of pests are influenced not only by different wavelengths of light but also by crop varieties. Different crops attract different types of pests, as shown in **Figure 3**, the pests of food crops and economic crops.
- ***Natural enemies*** Biological control is a method which utilize natural enemies of pests for control. Natural enemies and pests have the characteristics of interdependence and mutual control. The introduction of natural enemies can be beneficial to controlling the population density of pests and achieving effective control. Therefore, the number of natural enemies has a greater impact on the phototactic rhythm of pests.

The above analysis shows that pest phototropic rhythm is a physiological behavior influenced by the synergistic effect of many factors.

## 4 Study on the phototactic rhythm of pests

Crops are mainly divided into food crops and economic crops. Generally, the various pests have a different phototactic rhythm which is affected by meteorological, insecticidal devices, physiological status, and other factors. **Table 4** shows that the phototactic rhythm of pests have been studied in different research.

**Table 4:** The phototactic rhythm of main pests in different crops.

Paper	Research time	Research place	Sample area	Research crops	Type of lamp	Main pest	Phototactic rhythm of pests
(He, 2019)	2016-2018	Guangxi	N/A	Rice	Search lamp	*Sogatella Furcifera* and *Nilaparvata Lugens Stal*	18:00-19:00 5:00-6:00
(Cai et al., 2016)	2010-2011	Shandong	N/A	Rice and maize	Vibration frequency insecticidal lamp	*Laodelphax Striatellus*	18:00-19:00
						*Sogatella Furcifer*	5:00-6:00
(Qi et al., 2014)	2012-2013.4-10	Guangdong	0.7hm^2^	Rice	Search lamp	*Cnaphalocrocis Medinalis*	1:00-5:00
(Yang et al., 2014)	2012-2013	Jiangsu	2.4hm^2^	Rice	LED trap lamp	*Sogatella Furcifera* and *Nilaparvata Lugens Stal*	18:00-19:00 5:00-6:00
(Yang et al., 2012)	2011.5-6	Guangdong	12hm^2^	Rice, vegetable and maize	Intelligent solar insecticidal lamp	*Chilo Suppressalis*	23:00-1:00 3:00-5:00
						*Cnaphalocrocis Medinalis*	20:00-21:00 1:00-3:00
						*Sogatella Furcifera*and *Nilaparvata Lugens Stal*	20:00-21:00 21:00-4:00
						*Ostrinia Furnacalis*	19:00-22:00 00:00-2:00
						*Plutella Xylostella*	20:00-21:00 22:00-3:00
						*Spodoptera Litura*	00:00-3:00
(Zhou, 2007)	2007.4-8	Sichuan	N/A	Rice	Intelligent solar insecticidal lamp	*Chilo Suppressalis*	21:00-23:00 2:00-4:00
(Zhang and Huang, 2005)	2004	Chongqing	10hm^2^	Rice	Vibration frequency insecticidal lamp	*Chilo Suppressalis*	20:00-23:00 3:00-4:00
(Gu et al., 2004)	1999-2003	Jiangsu	N/A	Cotton	Double-waves trapping lamp	*Hymenia Recurvalis*	19:00-20:00 1:00-4:00
						*Holotrichia Perllela*	19:00-20:00
						*Gryllotalpa Africana*	20:00-22:00 22:00-00:00
						*Cifuna Locuples Walker*	20:00-21:00 4:00-5:00
						*Naranga Aenescens*	22:00-00:00 0:00-1:00
						*Conogethes Punctiferalis*	19:00-20:00
						*Cnaphalocrocis Medinalis*	18:00-19:00 2:00-5:00
(Yang, 2014)	2011-2012 6-7	Beijing,Hebei	N/A	Cotton and vegetable	Search lamp	*Ostrinia Furnacalis*	21:00-23:00
						*Heliothis Armigera*	21:00-23:00 3:00-4:00
						*Loxostege Sticticalis*	22:00-24;00
						*Mythimna Seperata*	1:00-4:00
(Tu et al., 2020)	2019-2020.5	Jiangxi	40hm^2^	Tea	Intelligent solar insecticidal lamp	*Ectropis Obliqua Hypulina Wehrli*	19:00-1:00
						*Anomala Corpulenta Motschulsky*, *Aeolesthes Induta Newman*	19:00-24:00
						*Empoasca Pirisuga Matumura*	18:00-20:00 5:00-7:00
						*Euproctis Pseudoconspersa Strand*	19:00-23:00
(Tu et al., 2018)	2016-2017	Jiangxi	2hm^2^	Tea	LED solar insecticidal lamp	*Ectropis Obliqua Hypulina Wehrli*	19:00-24:00
						*Anomala Corpulenta Motschulsky*, *Aeolesthes Induta Newman*	18:00-24:00
						*Empoasca Pirisuga Matumura*	17:30-19:30 05:00-07:30
(Wan et al., 2008)	2006.8.30-9.3	Shanghai	17.2hm^2^	Vegetable	Vibration frequency insecticidal lamp	*Euproctis* *Pseudoconspersa Strand* *Spodoptera Litura*, *Spodoptera Exigua*, *Plutella* *Xylostella*,*Spoladea Recurvalis*	19:30-24:00 19:00-24:00
(Tu et al., 2016)	6.1-8.16	Jiangxi	40 hm^2^	Vineyard	LED solar insecticidal lamp	*Agrotis Ypsilon*	19:00-22:00 23:00-03:00
						*Gryllotalpa Orientalis Burmeister*	21:00 -23: 30
						*Spodoptera Exigua*	18:00-20:30 (female) 23:00-3:00(male)
						*Spodoptera Litura*	19:0 - 21:30 (female) 23:00-3:00 (male)
						*Spoladea Recurvalis*	19:00-21:00
						*Anomala Corpulenta Motschulsky*	20:00-23:00
						*Empoasca Flavescens*	20:00-23:00
						*Apolygus Lucorum*	19:00-21:00 00:00-03:00

The symbol "N/A" means that the content of this column is not described by this literature, which is not clear whether it contains or specific values.

### 4.1 Phototactic rhythm of pests in food crops

#### 4.1.1 Phototactic rhythm of pests in maize

Maize, one of the major food crops in China, plays an important role in Chinese daily life. However, outbreaks of pests such as *Mythimna Seperata*, *Proxenus Lepigone*, and *Ostrinia Furnacalis* have caused serious economic losses to the yield and quality. *Ostrinia Furnacalis* has become the greatest dangerous pest in maize cultivation. Consequently, the research on the phototropic rhythm of *Ostrinia Furnacalis* is beneficial for improving SIL pest management in maize.

**Figure 4:**
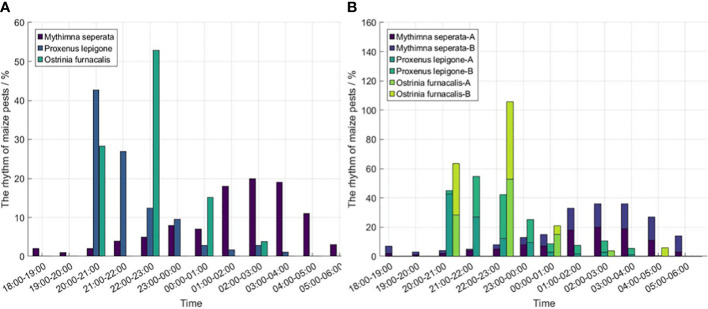
The phototactic rhythm of maize pests. **(A)** The phototactic rhythm of maize pests during the same period, **(B)** The cumulative phototactic rhythm of maize pests in different periods. *(Mythimna Seperata* A - 2014, *Mythimna Seperata* B - 2015, *Proxenus Lepigone* A - 2011.5.9, *Proxenus Lepigone* B - 2012.7.20, *Ostrinia Furnacalis* A - 2012.8.14, *Ostrinia Furnacalis* B - 2012.8.16).

**Figure 4A** shows that *Mythimna Separata* take off within half an hour of sunset and reached their highest numbers after midnight. It confirms the *Mythimna Separata* are more active in the morning (Zhang et al., 2017). On the contrary, the phototactic rhythm of *Proxenus Lepigone* and *Ostrinia Furnacalis* are mainly concentrated before midnight. Specifically, **Figure 4B** shows that the phototactic rhythm of *Mythimna Separata* mainly take place at 1:00 - 4:00 and 1:00 - 5:00 in 2014 and 2015, respectively. *Mythimna Separata* has the same phototropic rhythm in 2014 and 2015, and are both more active after midnight. In addition, The time differences in sunset have an effect on the phototactic rhythm of pests (Zhang et al., 2013). In general, the phototactic rhythm of pests vary with sunset time. For example, the sunset in July is delayed than that in May, so the phototactic rhythm of *Proxenus Lepigone* took place at 20:00 - 22:00 (*Proxenus Lepigone*-A) and 21:00 - 00:00 (*Proxenus Lepigone*-B) in May and July, respectively. The phototactic rhythm of *Ostrinia Furnacalis* is relatively consistent, which is concentrated in the middle night (20:00 - 00:00), as shown in **Figure 4B**.

#### 4.1.2 Phototactic rhythm of pests in rice

Rice plays an important part in Chinese food crops. Rice is affected by many pests, mainly including *Cnaphalocrocis Medinalis*, *Nilaparvata Lugens Stal*, and *Chilo Suppressalis*, etc. The phototactic rhythm of rice pests are influenced by all kinds of factors in nature.

**Figure 5:**
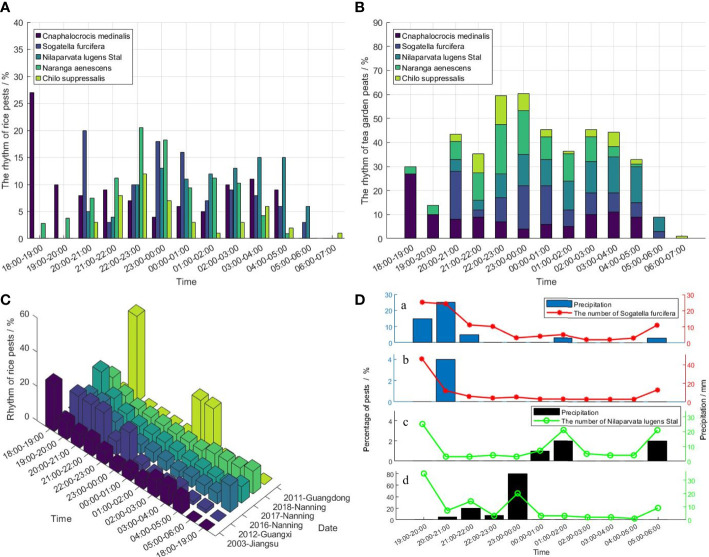
The phototactic rhythm of rice pests. **(A)** The phototactic rhythm of Cnaphalocrocis Medinalis, Sogatella Furcifera, Nilaparvata Lugens Stal, Naranga Aenescens, Chilo Suppressalis in rice crops, **(B)** The bar chart of Rhythm for Cnaphalocrocis Medinalis, Sogatella Furcifera, Nilaparvata Lugens Stal, Naranga Aenescens, Chilo Suppressalis in rice crops, **(C)** The phototactic rhythm of Cnaphalocrocis Medinalis in different regions and years, **(D)** The effects of Precipitation on rice pests, (a) The effect of precipitation on Sogatella Furcifera on June 15, (b) The effect of precipitation on Sogatella Furcifera on June 16, (c) The effect of precipitation on Nilaparvata Lugens Stal on June 24, (d) The effect of precipitation on Nilaparvata Lugens Stal on June 28 (Yang, 2014).

**Figure 5A** shows that the phototactic rhythm of rice pests is more active throughout the night. The *Cnaphalocrocis Medinalis* began to take off after sunset (Gu et al., 2004; Qi et al., 2014), and then the peaked phototactic rhythm of hCnaphalocrocis Medinalis mainly concentrates during the 19:00 - 21:00. The flight distance of *Cnaphalocrocis Medinalis* is affected by its physical strength and the external environment. (Yang et al., 2012; Yang, 2014) found that the adult of *Sogatella Furcifera* and *Nilaparvata Lugens Stal* can reach the peak value in the morning and night. The phototactic rhythm of *Naranga Aenescens* and *Chilo Suppressalis* mainly occurred before midnight (Gu et al., 2004; Zhou, 2007), as shown in **Figure 5B**. Those studies (Gu et al., 2004; Qi et al., 2014; He, 2019) have shown that *Cnaphalocrocis Medinalis* mainly took place before midnight. However, the phototropic rhythm of the *Cnaphalocrocis Medinalis* changed with the change of location. as shown in **Figure 5C**, the *Cnaphalocrocis Medinalis* in Guangxi in 2011 peaked at midnight (Yang et al., 2012). Therefore, the phototactic rhythm of pests are affected by various time and location.

(Yang, 2014) studied the effect of the precipitation on the take-off behavior of *Sogatella Furcifera* and *Nilaparvata Lugens Stal*. **Figure 5D** shows that the *Sogatella Furcifera* and *Nilaparvata Lugens Stal* of phototactic rhythm mainly is morning-dusk bimodal rhythm. In addition, on the nights without precipitation, the amount of pest outbreaks at dusk is higher than in the morning. The rainfall nights, the phototactic rhythm of *Sogatella Furcifera* and *Nilaparvata Lugens Stal* often is a multi-peaked curve. Thus, the precipitation at night can promote the take-off of *Sogatella Furcifera* and *Nilaparvata Lugens Stal*. **Figure 5a**, and **Figure 5b**, show that the precipitation has less effect on *Sogatella Furcifera*. However, The data of **Figure 5c**, and **Figure 5d**, show that the precipitation has a greater effect on *Nilaparvata Lugens Stal* than *Sogatella Furcifera*.

### 4.2 The phototactic rhythm of pest in economic crops

At present, China has formed a high-quality and efficient production technology system for economic crops such as cotton, tea, vegetable gardens, and orchards (Wu et al., 2021). However, frequent outbreaks of pests reduce crop yields and limit the sustainability of green agricultural crops. Therefore, it is necessary to study the phototactic rhythm of pests in different economic crops (Yang et al., 2014).

#### 4.2.1 Phototactic rhythm of pests in cotton

**Figure 6:**
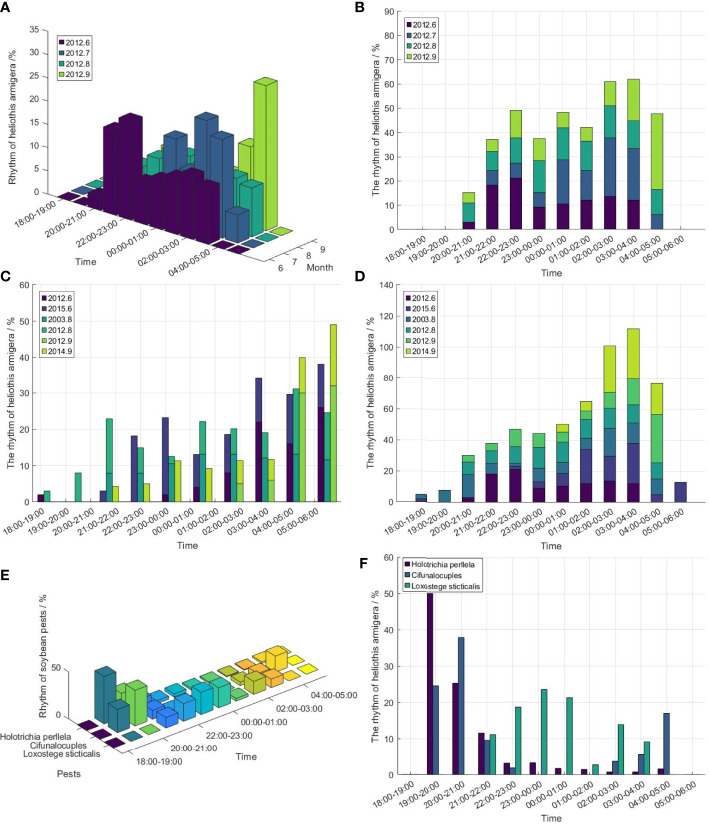
The phototactic rhythm of cotton pests. **(A)** The phototactic rhythm of *Helicoverpa Armigera* in Beijing from June to September 2012, **(B)** The cumulative phototactic rhythm of *Helicoverpa Armigera* in Beijing from June to September 2012, **(C)** The phototactic rhythm of *Helicoverpa Armigera* from June to September in different regions and years, **(D)** The cumulative phototactic rhythm of *Helicoverpa Armigera* from June to September in different regions and years. (2012.6 - Beijing, 2015.6 - Shandong, 2012.7 - Beijing, 2003.8 - Jiangsu, 2012.8 - Beijing, 2012.9 - Beijing, 2014.9 - Shandong) **(E)** The phototactic rhythm of *Holotrichia Perllela, Cifuna Locuples Walker, Loxostege Sticticalis* in cotton crops, **(F)** The bar chart of Rhythm for *Holotrichia Perllela, Cifuna Locuples Walker, Loxostege Sticticalis* in cotton crops.

Generally, *Helicoverpa Armigeras* often migrant at sunset and massively land at sunrise. Moreover, the migration speed and landing direction of *Helicoverpa Armigera* may have been influenced by the different climatic conditions (such as rain, airflow, etc.),. *Helicoverpa Armigera* was forced to fall and formed pest outbreak areas due to precipitation. At the same time, the area pointed by the wind direction may become the hazard area of *Helicoverpa Armigera* (Dingle and Drake, 2007). Due to the different lamps types, *Helicoverpa Armigera* may form a different phototactic rhythm, such as trap lamps in Jiangsu province, searchlights in Shandong and Beijing (Gu et al., 2004; Zhang, 2013; Zhang et al., 2017). As shown in **Figure 6A**, the phototactic rhythm of *Helicoverpa Armigera* mainly concentrated after midnight in the year 2012, Beijing, in addition to two rhythm peaks in June (The main peak of *Helicoverpa Armigera* was before midnight and the second peak was after midnight). However, the data in **Figure 6B** shows that the phototactic rhythm of the *Helicoverpa Armigera* happen after midnight. **Figure 6C** shows that the phototactic rhythm of *Helicoverpa Armigera* inJiangsu province was a bimodal form in August 2003, and the main peak and the secondary peak were mainly at 18:00 - 21:00 and 1:00 - 4:00, respectively. **Figure 6D** indicates that the *Helicoverpa Armigera* phototactic rhythm in other areas is mainly concentrated before midnight. It belonged to the type of single-peak phototactic rhythm. The main peak is mainly at 2:00 - 4:00 after midnight.

In addition to *Helicoverpa Armigera*, there are *Holotrichia Perllela*, *Cifuna Locuples Walker*, *Loxostege Sticticalis* can impact the yield of the cotton crop. As shown in **Figure 6E**, the phototactic rhythm of cotton pests is acted all night, whose peak mainly occurred before midnight. **Figure 6F** shows that the *Holotrichia Perllela* is mainly active between 19:00 and 21:00. The *Cifunalocuples* have two peak values, which the main peak is mainly between 20:00 and 21:00, and the secondary peak is relatively gentle during 4:00 - 5:00(the next day). In contrast, the phototactic rhythm of *Loxostege Sticticalis* mainly concentrated at midnight (22:00 - 1:00 (the next day)). Thus, the SIL in cotton crops needs to be turned on all night because the pests in crops are phototactic all night.

#### 4.2.2 Phototactic rhythm of pests in tea garden

With the rapid improvement of people’s living standards, drinking tea has become a part of leisure and entertainment in Chinese daily life. So, the demanding quality and quantity of tea are also increasing. Tea production is improved by using chemical pesticide methods to protect tea plantations from pest invasion. But chemical pesticide methods can cause serious environmental pollution and lower the tea quality (Lou et al., 2021). Therefore, it is urgent to adopt Physical methods to kill pests and improve the quality of tea leaves. Currently, SIL is the most widely used Physical method. The insecticidal working time of the SIL can be controlled according to the phototactic rhythm of pests in the tea garden, which is conducive to killing pests when there are many pests without producing pollution.

**Figure 7:**
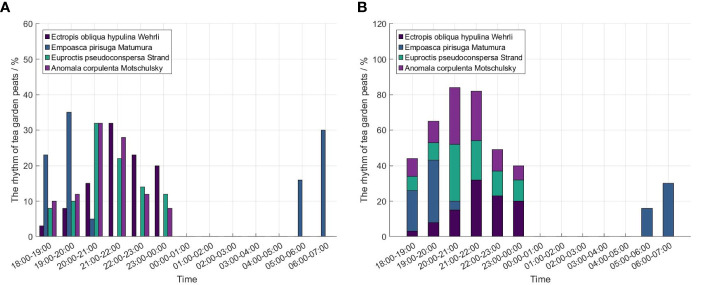
The phototactic rhythm of tea garden pests. **(A)** The phototactic rhythm of *Ectropis Obliqua Hypulina Wehrli, Empoasca Pirisuga Matumura, Euproctis Pseudoconspersa Strand, Anomala Corpulenta Motschulsky* in the tea garden, **(B)** The Cumulative chart of Rhythm for different pests in tea garden.

The main pests in tea garden include *Ectropis Obliqua Hypulina Wehrli*, *Empoasca Pirisuga Matumura*, *Anomala Corpulenta Motschulsky*, etc (Tu et al., 2018). The phototactic rhythm of the 20 - days tea garden pests are recorded by (Tu et al., 2018). The insecticidal working time of the SIL was 18:00 - 24:00 and 5:00 - 7:30. As shown in **Figure 7**, some pest adult (*Ectropis Obliqua Hypulina Wehrli*, *Euproctis Pseudoconspersa Strand* and *Anomala Corpulenta Motschulsky*) become active one hour after sunset and their phototactic rhythm mainly concentrate before midnight. However, the adult of *Empoasca Pirisuga Matumura* has two peak values. The phototactic rhythm of *Empoasca Pirisuga Matumura* adults was mainly around the evening (18:00 - 20:00) and 2 hours in the morning (5:00 - 7:00). Since the SILs in this investigation did not perform insecticidal work between 00:00 and 5:00, the above conclusions have some limitations and further experimental studies are needed.

**Table 5** shows that the amount of pests is influenced by different wavelengths in tea gardens (Tu et al., 2018). For example, the phototaxis wavelength of *Empoasca Pirisuga Matumura* was between 410 and 455 nm. However, the phototaxis wavelength of tea garden pest (*Ectropis Obliqua Hypulina Wehrli* and *Euproctis Pseudoconspersa Strand*) is 365 - 410 nm. The phototaxis wavelength of *Anomala Corpulenta Motschulsky* is 365 - 455 nm. Therefore, ultraviolet [(380 ± 20) nm] and blue light [(420 ± 20) nm] are better lamps to be used for pest management in the tea garden. In addition, SIL with different wavelengths and insecticidal working hours are selected to kill pests in tea gardens. It is beneficial to control pests and improve crop yield.

**Table 5:** Effect of wavelength on the phototactic rhythm of main pests in tea garden.

Main Pests	Month	Wavelength/nm	Rhythm of pests
*Ectropis Obliqua Hypulina Wehrli*	5-10	380-400	19:00-24:00
*Empoasca Pirisuga Matumura*	5-11	410-420	17:30-19:30, 05:00-07:30
*Euproctis pseudoconspersa Strand*	5-10	380-400	19:30-24:00
*Anomala Corpulenta Motschulsky*	5-9	380-450	18:00-24:00
*Aeolesthes induta Newman*	5-9	380-450	18:00-24:00

#### 4.2.3 Phototactic rhythm of pests in vegetable garden

**Figure 8:**
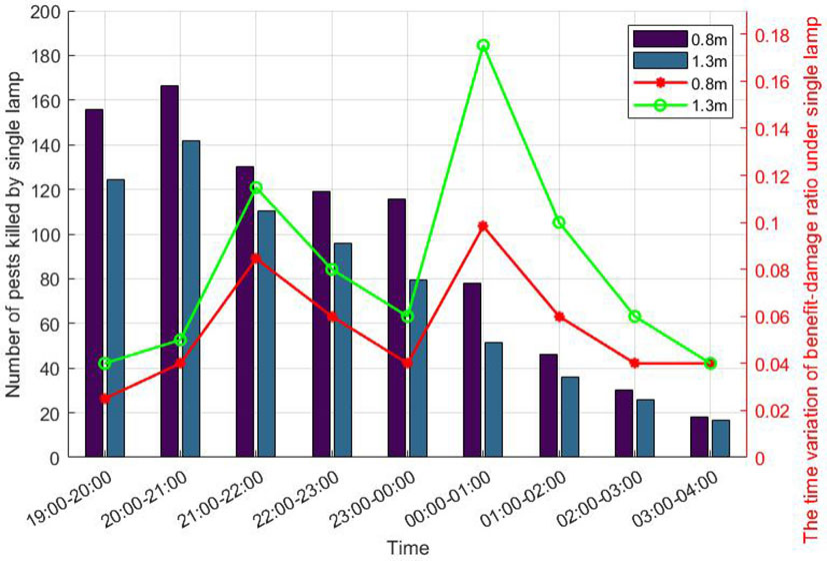
The phototactic rhythm of vegetable garden pests. The left label is the phototactic rhythm of vegetable pests, and the right label is the phototactic rhythm of SILs with different heights.

**Figure 8** shows that the major pests in the vegetable garden (mainly growing green leafy vegetables such as brussels sprouts, amaranth, spinach, and cabbage) are most active before midnight (Wan et al., 2008). Among them, *Plutella Xylostella* mainly have two peaks between 22:00 - 3:00 (the next day) and 20:00 – 21:00, respectively (Yang et al., 2012). (Xu et al., 2014) thought that the peak of *Hymenia Recurvalis* and *Anomala Corpulenta* were between 19:00- 21:00 and 20:00 - 23:00 respectively. In addition, the investigations (Wan et al., 2008) showed that there were differences in the phototactic rhythm of the natural and the artificial light of the pests. Moreover, the insecticidal effect of thelamp height of 0.8 meters is better than that of 1.3 meters.

#### 4.2.4 Phototactic rhythm of pests in orchard

The main pests in Chinese southern vineyards are *Agrotis Ypsilon*, *Gryllotalpa Orientalis Burmeister*, *Spodoptera Litura*, *Anomala Corpulenta Motschulsky*, and *Paranthrene Regalis Butler*, etc. The adoption of Physical methods in vineyards not only controls pests but also reduces the use of chemical pesticides, which is beneficial for ecological agriculture and economic benefits.

**Figure 9:**
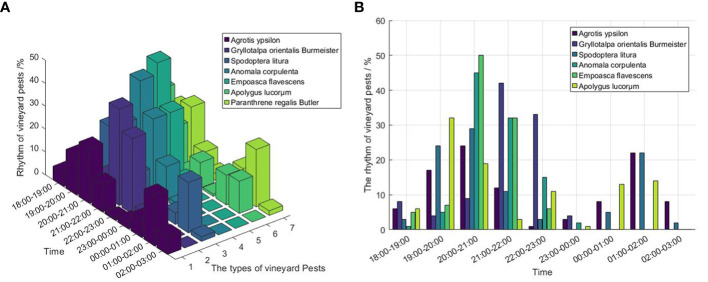
The phototactic rhythm of vineyard pests. **(A)** The phototactic rhythm of Agrotis Ypsilon, Gryllotalpa Orientalis Burmeister, Spodoptera Litura, Anomala Corpulenta Motschulsky, Empoasca Flavescens, Oriental Mole Crickets, Spodoptera Litura, Apolygus Lucorum and Paranthrene Regalis Butler in vineyard, **(B)** The bar chart of Rhythm for Agrotis Ypsilon, Gryllotalpa Orientalis Burmeister, Spodoptera Litura, Anomala Corpulenta Motschulsky, Empoasca Flavescens, Oriental Mole Crickets, Spodoptera Litura, Apolygus Lucorum and Paranthrene Regalis Butler in vineyard.

As shown in **Figure 9A**, the vineyard pests acted all night, and the phototactic rhythm of the main peak mainly concentrated before midnight. **Figure 9B** shows that the phototactic rhythm of *Agrotis Ypsilon* and *Spodoptera Litura* between 19:00 and 22:00 were the highest, *Anomala Corpulenta Motschulsky* and *Empoasca Flavescens* are the most active between 20:00 and 23:00, and *Gryllotalpa Orientalis Burmeister* and *Apolygus Lucorum* are most active between 19:00 and 23:00. However, the phototactic rhythm of *Paranthrene Regalis Butler* have two peaks, which were mainly between 19:00 - 21:00 and 1:00 - 2:00.

The phototactic rhythm of different pests varies greatly (Yang et al., 2012). The Coleoptera pests are 321 sensitive to 400 nm wavelength. The wavelength of 360 nm and 520 nm have the best trapping effect on 322 Lepidoptera pests and Hemiptera pests (Tu et al., 2016). As shown in **Table 6**, most vineyard pests (such as *Agrotis Ypsilon*, *Gryllotalpa Orientalis Burmeister* and *Paranthrene Regalis Butler*, etc.) are greatly influenced by the wavelength of 330 - 400 nm, except for the phototaxis of *Apolygus Lucorum* on the mixed light source of 550 - 590 nm. *Anomala Corpulenta Motschulsky*, *Empoasca Flavescens*, *Oriental Mole Crickets*, *Spodoptera Litura*, *Paranthrene Regalis Butler*, etc.) are greatly influenced by the wavelength of 330 - 400 nm. It is conducive to efficiently killing pests and protecting natural enemies by selecting different wavelengths of SIL.

**Table 6:** Effect of wavelength on the phototactic rhythm of main pests in vineyard.

Main Pests	Wavelength/nm	phototactic rhythm of pests
*Agrotis Ypsilon*	350-400	19:00-22:00, 23:00-03:00
*Gryllotalpa Orientalis Burmeister*	350-400	21:00-23:30
*Spodoptera Exigua*	550-590	18:00-20:30 (female), 23:00-03:00(male)
*Spodoptera Litura*	350-400	19:00-21:30 (female), 23:00-03:00(male)
*Hymenia Recurvalis Fabricius*	350-400	19:00-21:00
*Anomala Corpulenta Motschulsky*	350-400	20:00-23:00
*Empoasca Pirisuga Matumura*	350-400	20:00-23:00
*Apolygus Lucorum*	550-590	19:00-21:00, 00:00-03:00

## 5 Challenge issues and future directions

Every year crop production is getting damaged due to pest infestation. Although the phototactic rhythm of pests has been applied for SILs, it still faces many challenges.

### 5.1 The standard for green pest management

After more than a decade of research, the pest phototropic rhythms have a common pattern. Due to the differences in the crop growth environment and geographical regions, their phototropic rhythms may show minor errors. To improve the data quality, the pest phototactic rhythm data can be created into a new public database for access by research scholars. Thus, a uniform standard for green pest management needs to be established. Meanwhile, the research on physiological mechanism differences between pests and natural enemies at different wavelengths will be conducive to reducing the rate of killing natural enemies. The phototactic rhythm of different pests to specific wavelengths contributes to the intelligent monitoring and control of agricultural pests by SILs.

### 5.2 Intelligent system of SILs

At present, the research on the phototactic rhythm of pests are based on manual counting (Zhang et al., 2017), which is time-consumed and less intelligent. In addition, the majority SILs adopt remote techniques to regulate the SIL kill durations. The long insecticide working time of SILs may cause low energy utilization. Based on the above analysis, the low efficiency and energy waste of SILs can be solved by improving the intelligent system of SILs.

### 5.3 New technologies and devices

Currently, the methods of pest monitoring mainly include sound signals, radar, remote sensing technology, pest monitoring, trap lamp with various sensors and Unmanned Aerial Vehicles (UAVs) with remote sensing. Although these methods have achieved excellent results, they are expensive and not suitable for large-scale deployment. Therefore, it is necessary to cooperate with radar and pest monitoring lamps to manage pests and reduce costs. Building a low-cost, wide-range, flexible and controllable green management system and monitoring device is essential.

## 6 Conclusion

In this paper, we present a comprehensive survey on the phototactic rhythm of pests in smart agriculture and phytoprotection. The research on pest habits is investigated and the distribution of pests at different time periods is outlined. Then, the current challenge issues of green pest management are analyzed in the context of pest phototactic rhythm. Finally, future research directions are envisioned. This paper benefits agricultural researchers with a comprehensive understanding of the photosynthetic rhythm of pests. In addition, the application of pest phototactic rhythm for SILs still faces great challenges and issues of intelligence and precision. Future research directions should include addressing these challenges and exploring them in conjunction with emerging technologies.

## Author contributions

HY, LS and YY participated in the conception of the experimental idea. HY performed the platform construction and simulation experiments. HY, LS, FY, YJ and YY wrote and revised the paper. All authors contributed to the article and approved the submitted version.
